# The association between spinal health and visual function in a pediatric population: insights from large-scale health examinations

**DOI:** 10.3389/fpubh.2025.1702548

**Published:** 2026-01-21

**Authors:** Jun Luo, Linhui Xie, Yulin Luo, Zili Cai, Xilang Wang, Chanyuan Wang, Tong Wang, Yu Tian, Aoxiang Wang

**Affiliations:** 1 Department of Ophthalmology, The Affiliated Children's Hospital of Xiangya School of Medicine, Central South University (Hunan Children’s Hospital), Changsha, Hunan, China; 2 Clinical Research Center for Pediatric Eye Diseases in Hunan Province, Changsha, Hunan, China; 3 Department of Children's Healthcare, The Affiliated Children's Hospital of Xiangya School of Medicine, Central South University (Hunan Children’s Hospital), Changsha, Hunan, China; 4 Pediatrics Research Institute of Hunan Province, The Affiliated Children’s Hospital of Xiangya School of Medicine, Central South University (Hunan Children’s Hospital), Changsha, Hunan, China; 5 Changsha Aier Eye Hospital, Changsha, Hunan, China

**Keywords:** pediatric population, physical examination, spherical equivalent, spinal health, stereopsis

## Abstract

**Background:**

Physical examinations are critical for early detection of spinal abnormalities and visual function issues in children. Spinal health has a significant impact on both physical and psychological well-being, while untreated abnormalities may lead to postural deformities. Despite evidence linking spinal posture and balance control to visual function, the relationship between these factors remains underexplored. This study investigates the association between spinal health and visual function.

**Methods:**

Data were collected from 875 children undergoing health examinations. Spinal parameters, including the thoracic kyphosis angle and the angle of trunk rotation, were measured alongside visual and perceptual examinations and refractive status. Statistical analyses were performed to assess their relationships.

**Results:**

Significant differences were observed in the distribution of spinal parameters across three orders of stereopsis and spherical equivalent (SE). Specifically, the median angle of trunk rotation (ATR) was 2° (IQR: 1°–3°) in participants with level 4 zero-order stereopsis, which was significantly lower than that in level 0 participants (median: 4°, IQR: 3°–5°, *p* < 0.05). The median kyphosis angle (KA) was 27° (IQR: 25°–29°) in those with pass-grade first-order stereopsis, significantly lower than the 35° (IQR: 32°–38°) in non-passers (*p* < 0.001). Spearman’s rank correlation analysis revealed a significant negative correlation between SE and both KA (rs = −0.18, *p* < 0.001) and ATR (rs = −0.32, *p* < 0.001). Additionally, gender differences were found in KA distribution, with females having a higher median KA (29°, IQR: 26°–31°) than males (27°, IQR: 25°–30°, *p* < 0.05).

**Conclusion:**

These findings suggest a potential link between spinal health and visual development. In this cross-sectional sample, poorer stereopsis and more myopic refractive error were associated with modestly higher kyphosis and trunk-rotation angles. These findings provide preliminary evidence for the link between visual function and spinal alignment in school-aged children. Incorporating spinal health assessments into pediatric visual screening programs could facilitate early intervention for both spinal and visual abnormalities, improving overall child health outcomes.

## Background

Physical examinations are integral to child health management, providing critical opportunities for the early detection and prevention of various conditions. These evaluations can play a pivotal role in ensuring spinal health and posture fitness, enabling the early identification of abnormalities. Adolescent idiopathic scoliosis (AIS) is a common three-dimensional spinal deformity that typically manifests between the ages of 10 and 18 years, affecting approximately 0.93 to 12% of the global pediatric population (1–3). Rapid progression of scoliosis during the adolescent growth spurt can lead to various cosmetic concerns, back pain, psychological issues, cardiopulmonary difficulties, and a decreased quality of life-related to health (4, 5). Early diagnosis and intervention are crucial for preventing further progression of the curvature and avoiding the need for invasive treatments.

Visual function, particularly stereopsis and refractive status, is also critical for pediatric development. Stereopsis, the ability to perceive three-dimensional depth, develops rapidly in children aged 4–6 years and stabilizes by 10 years, with abnormalities reported in 7% of school-age children. Refractive errors, including myopia and hyperopia, are highly prevalent in pediatric populations, with myopia affecting nearly 55% of Chinese children aged 6–16 years (6–8). Untreated visual function impairments can disrupt postural balance, as postural control relies on the integration of visual, vestibular, and proprioceptive inputs (9).

Existing research predominantly focuses on the relationship between established spinal deformities and visual impairment, with limited studies investigating early-stage spinal abnormalities (mild kyphosis or trunk rotation without full-blown scoliosis) and their association with visual function in children (10, 11). Additionally, the bidirectional interaction between visual function and spinal health remains underexplored, especially in population-based health screenings (12).

This study aims to investigate the association between spinal health and visual function (stereopsis and refractive status) in children and adolescents aged 6–16 years during routine health screenings. We hypothesize that better stereopsis and more favorable refractive status are associated with improved spinal alignment. The findings are expected to inform comprehensive pediatric health screening strategies, facilitating early intervention for both conditions.

## Methods

### Patients and methods

#### Patients

A cross-sectional study investigated spinal curvature, visual acuity, refractive error, and binocular visual function (hereafter referred to as “visual function”) among children and adolescents aged 6–16 years who underwent a health checkup at our hospital during the summer vacation in July and August 2024. The inclusion criteria were: (1) children with no history of major visual or spinal disorders; (2) children willing to undergo the full spectrum of spinal and visual assessments. The exclusion criteria were as follows: (1) inability to cooperate or refusal to undergo examinations; (2) diagnosis of amblyopia, strabismus, or a history of ocular trauma or surgery upon ophthalmologic examination; (3) refractive spherical equivalent (SE) exceeding +3.00 D or less than −6.00 D in either eye; (4) diagnosed as systemic diseases affecting visual or refractive development; and (5) spinal and thoracic deformities, musculoskeletal abnormalities, or history of treatment.

The study protocol was approved by the Ethics Committee of the Hunan Children’s Hospital (Approval No.: YK2024-32). The study adhered to the principles of the Declaration of Helsinki. All participants and their guardians were informed of the purpose and methods of the study and voluntarily signed informed consent forms.

### Ocular examination

#### Refractive status assessment

All participants underwent a comprehensive history-taking process followed by a best-corrected visual acuity assessment using a Snellen chart and a prism test to exclude amblyopia and strabismus. A professional optometrist performed refractive measurements using an automatic refractometer (Topcon KR-8800; Topcon, Tokyo, Japan) and retinoscopy without pupil dilation to identify the best visual acuity. The SE was calculated as the sum of the spherical power and half of the cylindrical error.

#### Stereopsis assessment

Visual and perceptual examinations were conducted using a Windows 10-based PC, an LG2342p polarized 3D monitor (1920 × 1,080 resolution, 120 Hz refresh rate), and 3D polarized glasses. The examination utilized a visual-perceptual evaluation system developed by the National Engineering Research Center for Healthcare Devices, with stimulus templates generated using MATLAB. All assessments were performed under bright ambient lighting conditions, and participants wore their habitual spectacle corrections and 3D glasses; participants without refractive error or non-glass wearers were tested without additional correction. The 3D monitor was positioned such that the center of the screen was aligned horizontally and vertically with the participants’ eyes at a viewing distance of 80 cm, as described before (13).

**Figure 1:**
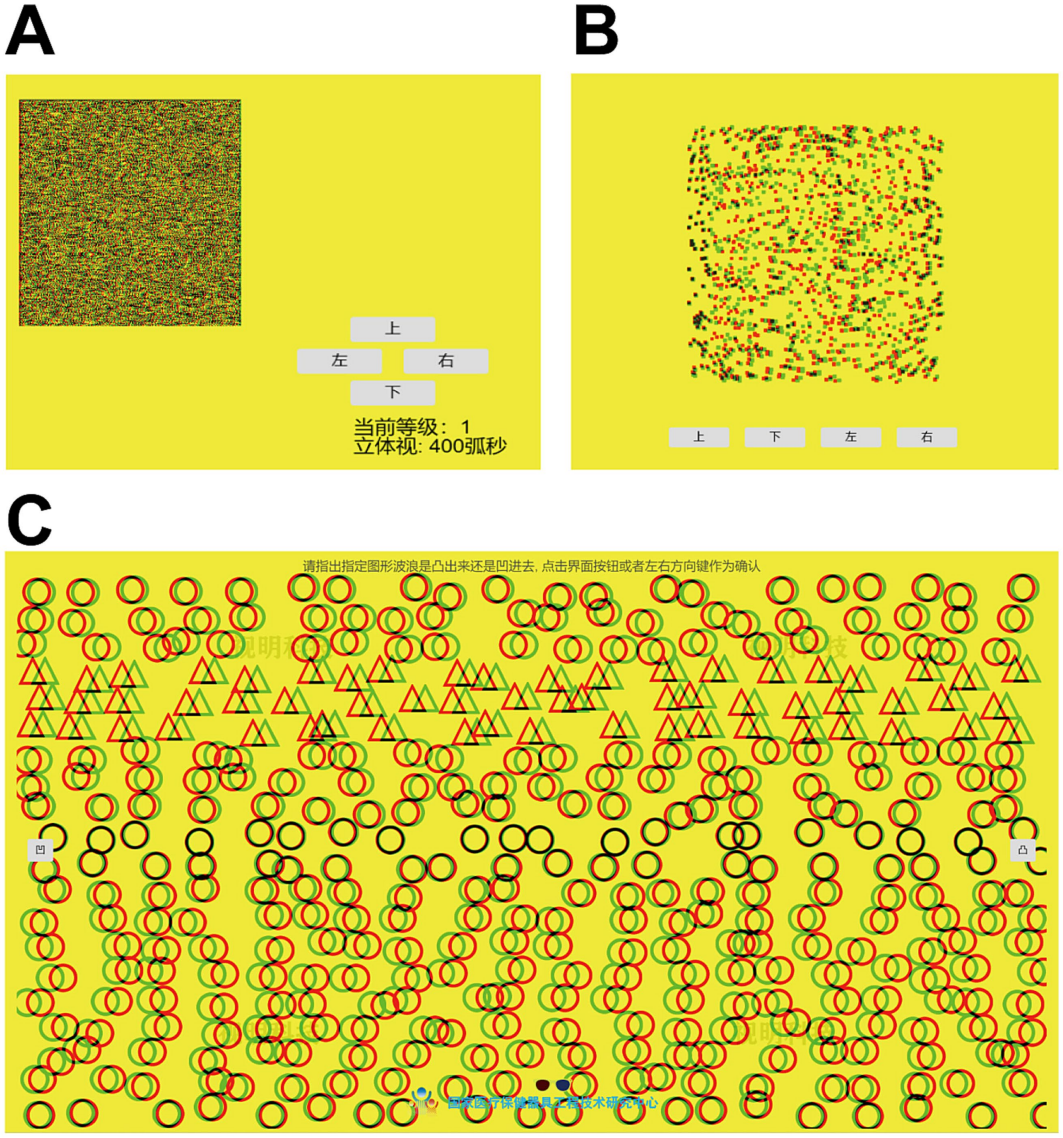
Measurement of stereopsis. **(A–C)** Evaluation of zero-order, first-order, and second-order stereopsis.

The stereopsis assessment consisted of zero-order, first-order, and second-order stereopsis, with the inspection sequence progressing from second-order to zero-order stereopsis (Figure 1).

1. Zero-order Stereopsis: Participants, wearing polarized glasses, identified the orientation of the “E” symbol in the images using keyboard arrow keys or interface buttons. Four images were shown with 400″, 300″, 200″, and 100″ disparities. The grading was as follows: 100″ = Level 4, 200″ = Level 3, 300″ = Level 2, 400″ = Level 1, and failure to recognize any = Level 0 (Figure 1A).
2. First-order Stereopsis: Participants, while wearing polarized glasses, identified the orientation of the “E” symbol against a high-speed moving background. The accuracy should be 100 percent to pass, which was recorded as “1,” any errors were recorded as “0” (Figure 1B).
3. Second-order Stereopsis: Participants determined whether the visual target appeared as a peak (convex) or valley (concave) with polarized glasses. Passing required 100% accuracy. The term “Pass” was recorded as “1,” while the term “Error” was recorded as “0” (Figure 1C).

### Spinal examination

#### The angle of trunk rotation

**Figure 2:**
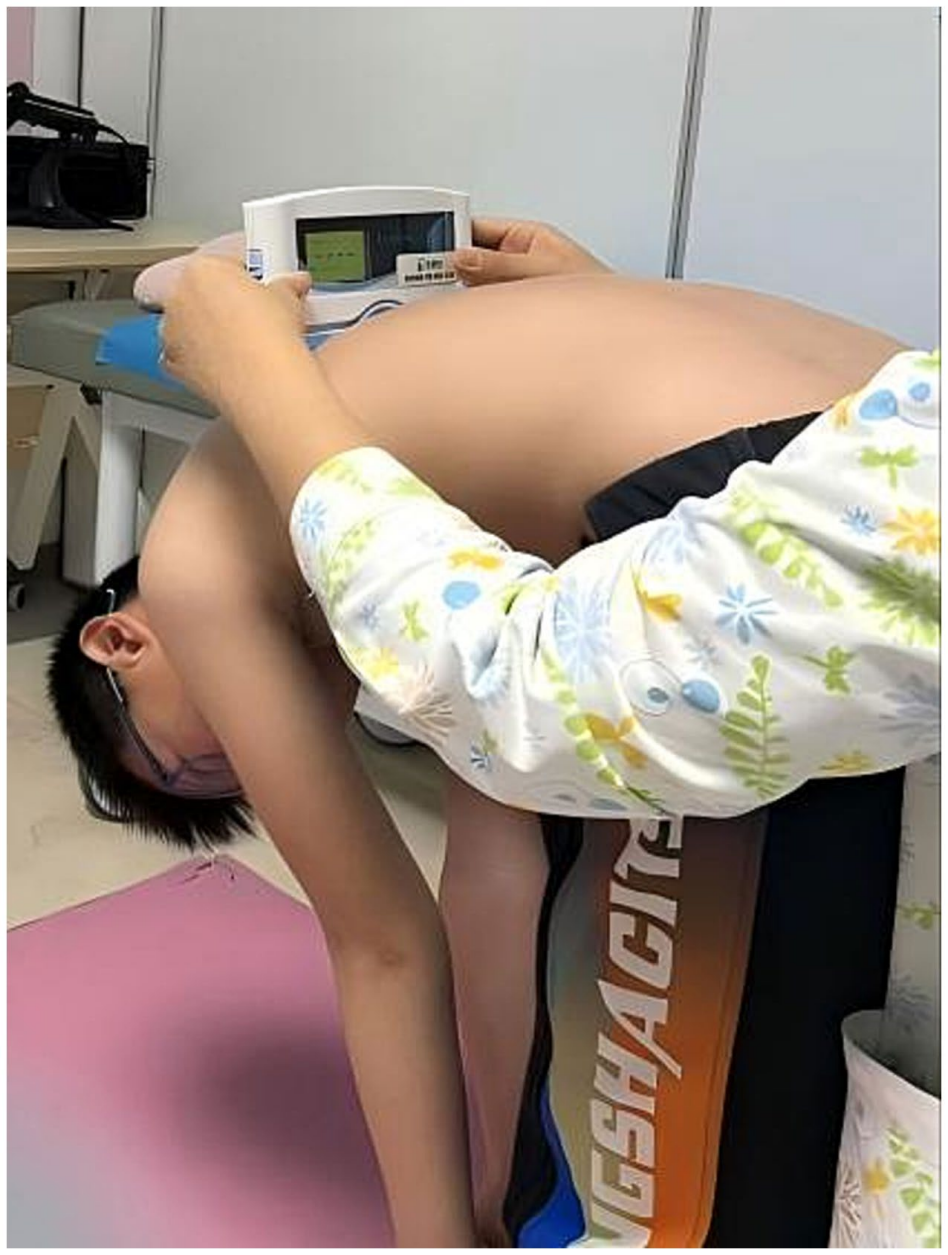
Posture of the participant during spinal parameter measurement. Participants should expose the spine on the back and stand barefoot with feet shoulder-width apart, palms pressed together, fingers aligned, and arms extended straight forward while bending at the trunk.

Experienced examiners measured the angle of trunk rotation (ATR) using the SpineScan system (OrthoScan Technologies, Rosh Pina, Israel). The procedure involved the following steps (14, 15): Participants should expose the spine on the back and stand barefoot with feet shoulder-width apart, palms pressed together, fingers aligned, and arms extended straight forward while bending at the trunk (Figure 2). Participants maintained their shoulders and hips in a horizontal position, their knees straight, and their legs as vertical as possible. The device was placed vertically aligned with the C7 spinous process and slid slowly down the back along the spinal midline to the L5 vertebra while ensuring constant speed and gentle contact without applying excessive pressure. The procedure was repeated three times, and the average trunk inclination angle was recorded. If the ATR exceeded 5°, participants and their guardians were advised to seek further evaluation from a spine surgery specialist.

### Spinal kyphosis

The spinal kyphosis angle (KA) was measured using the SpineScan system. Participants stood barefoot, with their feet shoulder-width apart and their arms extended forward at shoulder height. The device’s top midpoint was aligned with the C7 vertebra, and the instrument was gently pressed against the participant’s back. The device was slid horizontally along the spinal curve to the S1 vertebra while maintaining a constant speed and ensuring the bottom groove was aligned with the spinous processes without applying excessive pressure. Measurements were repeated three times, and the average kyphosis angle was recorded. If the kyphosis angle exceeded 40°, participants and their guardians were advised to seek further evaluation from a spine surgery specialist.

### Sample size calculation

Sample size was estimated using G*Power 3.1.9.7 software,^1^ based on the primary objective of detecting associations between visual function and spinal parameters. A conservative correlation coefficient (*r* = 0.15) was predefined. For calculation, we set a two-tailed significance level (*α*) of 0.05 and statistical power (1−*β*) of 0.8. Using a bivariate correlation model, the minimum required sample size was estimated to be 800. Considering potential incomplete data, 857 eligible children (6–16 years old) completed all assessments, which met the statistical power requirement.

### Statistical analysis

The SE was calculated as the sum of the spherical power and half of the cylindrical power, using data from the right eye for analysis. In this study, the independent variables were spinal parameters, specifically the thoracic KA and ATR, as well as refractive status (spherical equivalent). The dependent variables (outcomes) included visual function, specifically stereopsis (measured by zero-order, first-order, and second-order stereopsis), and other aspects of visual health, such as visual acuity. These measures were chosen based on their established relevance in assessing both spinal health and visual function, particularly in pediatric populations.

The normality of the data distribution was assessed using the Kolmogorov–Smirnov test. Age was presented as the mean ± standard deviation (SD), while other continuous variables were expressed as median and interquartile range (IQR), with IQR defined as the range between the 25th percentile (Q1) and the 75th percentile (Q3).

The following statistical methods were employed: the Mann–Whitney U test for non-normally distributed continuous variables, and Spearman’s rank correlation test for assessing relationships between non-parametric variables. Spearman’s rank correlation coefficient (r_s_) was used as a non-parametric measure of correlation. The Kruskal-Wallis H test was applied to compare differences among three or more independent groups for non-normally distributed continuous variables. If a significant difference was found (*p* < 0.05), post-hoc pairwise comparisons were conducted using Dunn’s test with a Bonferroni correction to identify which specific groups differed significantly. The significance level for post-hoc comparisons was adjusted to control for multiple comparisons.

Multivariable linear regression models were used to assess the independent associations of visual function (stereopsis grades) and refractive status (SE) with KA and ATR, respectively. Potential confounding factors including age and sex were adjusted in all models. Collinearity among variables was evaluated using variance inflation factor (VIF), with VIF < 2 indicating no significant multicollinearity.

All statistical analyses were performed using SPSS Statistics for Windows (version 26.0; IBM Corp., Armonk, NY, USA). A *p*-value of less than 0.05 was considered statistically significant. Detailed results of the multivariable regression models are presented in Supplementary Tables S1, S2.

## Result

### Participants’ baseline characteristics

A total of 857 children aged 6–16 years were included in this study, with a mean age of 9.42 ± 2.63 years. Among them, 512 (59.74%) were male and 345 (40.26%) were female, and no significant difference in age was observed between sexes (*p* > 0.05). The median SE of the right eye for all participants was −0.25 D (IQR: −1.75 to 0.38 D), with no significant difference between sexes. The distribution of stereoscopic function levels (0–2) is detailed in Table 1, with the majority of participants demonstrating relatively better stereoscopic function. The median spinal kyphosis angle was 28° (IQR: 25°–31°), and the median ATR was 3° (IQR: 2°–4°). Notably, 9 children were identified with a kyphosis angle exceeding 40°, among whom 6 had an ATR greater than 5°. These children were advised to seek further evaluation in a spine specialty clinic. Further details on the participants’ baseline characteristics are presented in Table 1.

**Table 1:** Participants’ characteristics.

Characteristic	Total	Male	Female
Age, mean years, (SD)	9.42 (2.63)	9.50 (2.63)	9.46 (2.62)
Sex (male), number (%)	857 (100)	512 (59.74)	345 (40.26)
SE, median (IQR)	−0.25 (−1.75–0.38)	−0.25 (−1.88–0.38)	−0.25 (−1.75–0.38)
Zero-order stereopsis, number (%)			
4	332 (38.74)	200 (23.34)	132 (15.40)
3	264 (30.81)	151 (17.62)	113 (12.19)
2	84 (9.80)	45 (5.25)	38 (3.55)
1	90 (10.50)	60 (7.00)	30 (3.50)
0	88 (10.26)	56 (6.53)	32 (3.73)
First-order stereopsis, number (%)			
Pass	749 (87.40)	444 (51.81)	305 (36.09)
Error	108 (12.60)	68 (7.93)	40 (4.67)
Second-order stereopsis, number (%)			
100	844 (98.48)	504 (58.81)	340 (39.67)
0	13 (1.52)	8 (0.93)	5 (0.59)
KA, median (IQR)	28 (25–31)	27 (25–30)	29 (26–31)
ATR, median (IQR)	3 (2–4)	3 (2–4)	3 (2–4)

### Relationship between spinal status and visual function

#### ATR and stereovision

**Figure 3:**
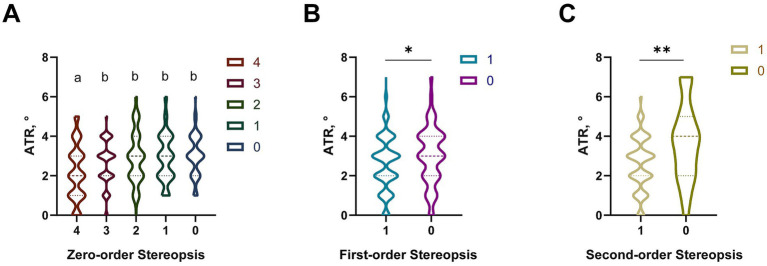
Distribution differences of the ATR among the three-level stereopsis function. **(A)** Distribution differences of the ATR among the 0-order stereopsis function groups. Statistical differences between groups were analyzed using the Kruskal-Wallis H test. Bars labeled with the same letter (e.g., a, b) are not significantly different, while bars labeled with different letters (e.g., a vs. b, ab vs. c) indicate significant differences (*p* < 0.05). **(B)** Distribution differences of the ATR between the two groups with the 1st-order stereopsis function. **(C)** Distribution differences of the ATR between the two groups with the 2nd-order stereopsis function. *p < 0.05, **p < 0.01, ***p < 0.001. ATR, the angle of trunk rotation.

In this study, we observed slight differences in the ATR distribution across the five levels of Zero-order Stereopsis. Sample sizes for each stereopsis group are shown in Table 1. As shown in Figure 3A, post-hoc analysis revealed that participants with level 4 zero-order stereopsis had a significantly lower median ATR (2°, IQR: 1°–3°) than those with levels 3 (3°, IQR: 2°–4°), 2 (3°, IQR: 2°–4°), 1 (4°, IQR: 3°–5°), and 0 (4°, IQR: 3°–5°) (all *p* < 0.05 after Bonferroni correction). No significant differences were found among levels 3, 2, 1, and 0 (*p* > 0.05).

For first-order stereopsis (Mann–Whitney U test, *Z* = −4.21, *p* < 0.001), participants who passed (*n* = 749) had a median ATR of 3° (IQR: 2°–3°), significantly lower than non-passers (*n* = 108, median: 4°, IQR: 3°–5°). The violin plot reveals that the ATR values for the non-passers were mostly concentrated at 4°, while those with “100% accuracy” had ATR values more concentrated around 2° and 3° (Figure 3B). For second-order stereopsis (Mann–Whitney U test, *Z* = −3.85, *p* < 0.001), passers (*n* = 844) had a median ATR of 3° (IQR: 2°–4°), while non-passers (*n* = 13) had a median ATR of 4° (IQR: 3°–6°) (Figure 3C).

#### Kyphosis and stereovision

**Figure 4:**
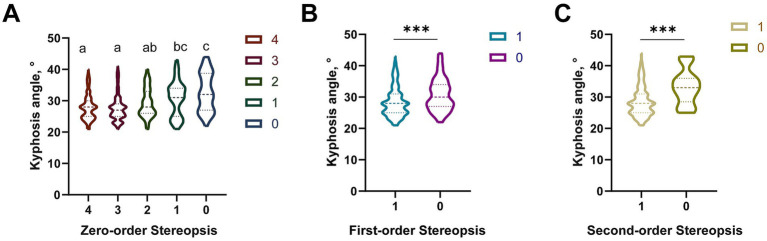
Distribution differences of the KA among the three-level stereopsis function. **(A)** Distribution differences of the KA among the 0-order stereopsis function groups. Statistical differences between groups were analyzed using the Kruskal-Wallis H test. Bars labeled with the same letter (e.g., a, b) are not significantly different, while bars labeled with different letters (e.g., a vs. b, ab vs. c) indicate significant differences (*p* < 0.05). **(B)** Distribution differences of the KA between the two groups with the 1st-order stereopsis function. **(C)** Distribution differences of the KA between the two groups with the 2nd-order stereopsis function. *p < 0.05, **p < 0.01, ***p < 0.001. KA, kyphosis angle.

Significant differences in KA distribution were observed across five levels of zero-order stereopsis (Kruskal-Wallis H test, *χ*^2^ = 35.72, *p* < 0.001) (Figure 4A). Participants with level 4 (median: 26°, IQR: 24°–28°) and 3 (median: 27°, IQR: 25°–29°) had significantly lower KA than those with level 1 (median: 32°, IQR: 29°–35°) and 0 (median: 33°, IQR: 30°–36°) (*p* < 0.001 after Bonferroni correction). KA of level 2 (median: 29°, IQR: 26°–31°) was significantly lower than that of level 0 (*p* < 0.01), but no difference was found between level 1 and 0 (*p* > 0.05).

For first-order stereopsis (Mann–Whitney U test, *Z* = −5.13, *p* < 0.001), passers (*n* = 749) had a median KA of 27° (IQR: 25°–30°), significantly lower than non-passers (*n* = 108, median: 35°, IQR: 32°–38°). For second-order stereopsis (Mann–Whitney U test, *Z* = −2.97, *p* < 0.01), passers (*n* = 844) had a median KA of 28° (IQR: 25°–31°), while non-passers (*n* = 13) had a median KA of 32° (IQR: 29°–34°) (Figures 4B,C).

#### Refraction and spinal status

**Figure 5:**
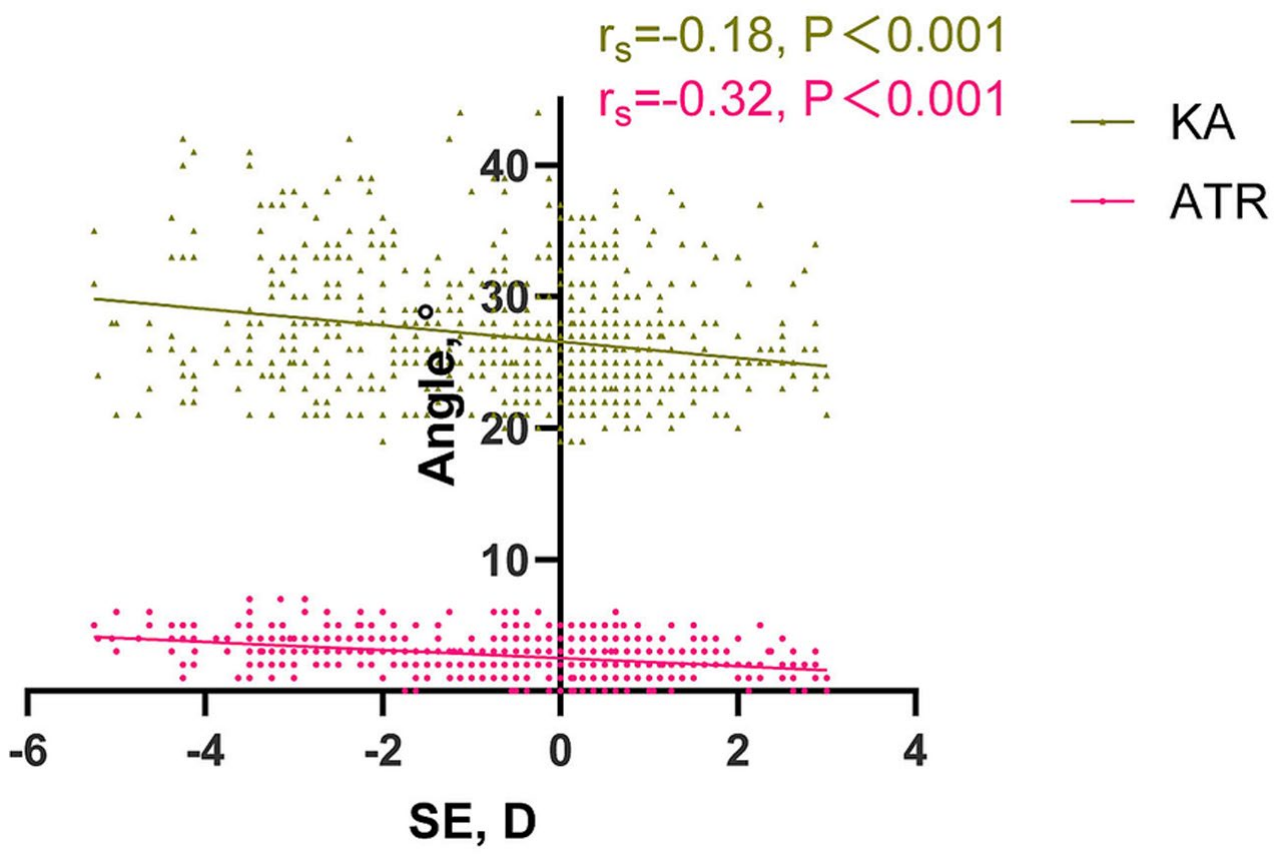
The relationship between SE, KA and ATR. SE and KA: rs = −0.18, *p* < 0.001; SE and ATR: rs = −0.32, *p* < 0.001. SE, spherical equivalent. ATR, angle of trunk rotation. KA, kyphosis angle.

In this study, we investigated the relationship between the SE and both KA and ATR (Figure 5). Spearman’s rank correlation analysis revealed a significant negative correlation between SE and both KA and ATR (r_s_ = −0.18, *p* < 0.001; r_s_ = −0.32, *p* < 0.001). Specifically, our findings indicate that as the SE approached a hyperopic state in the study participants, both KA and ATR values tended to be lower.

#### Gender and spinal status

**Figure 6:**
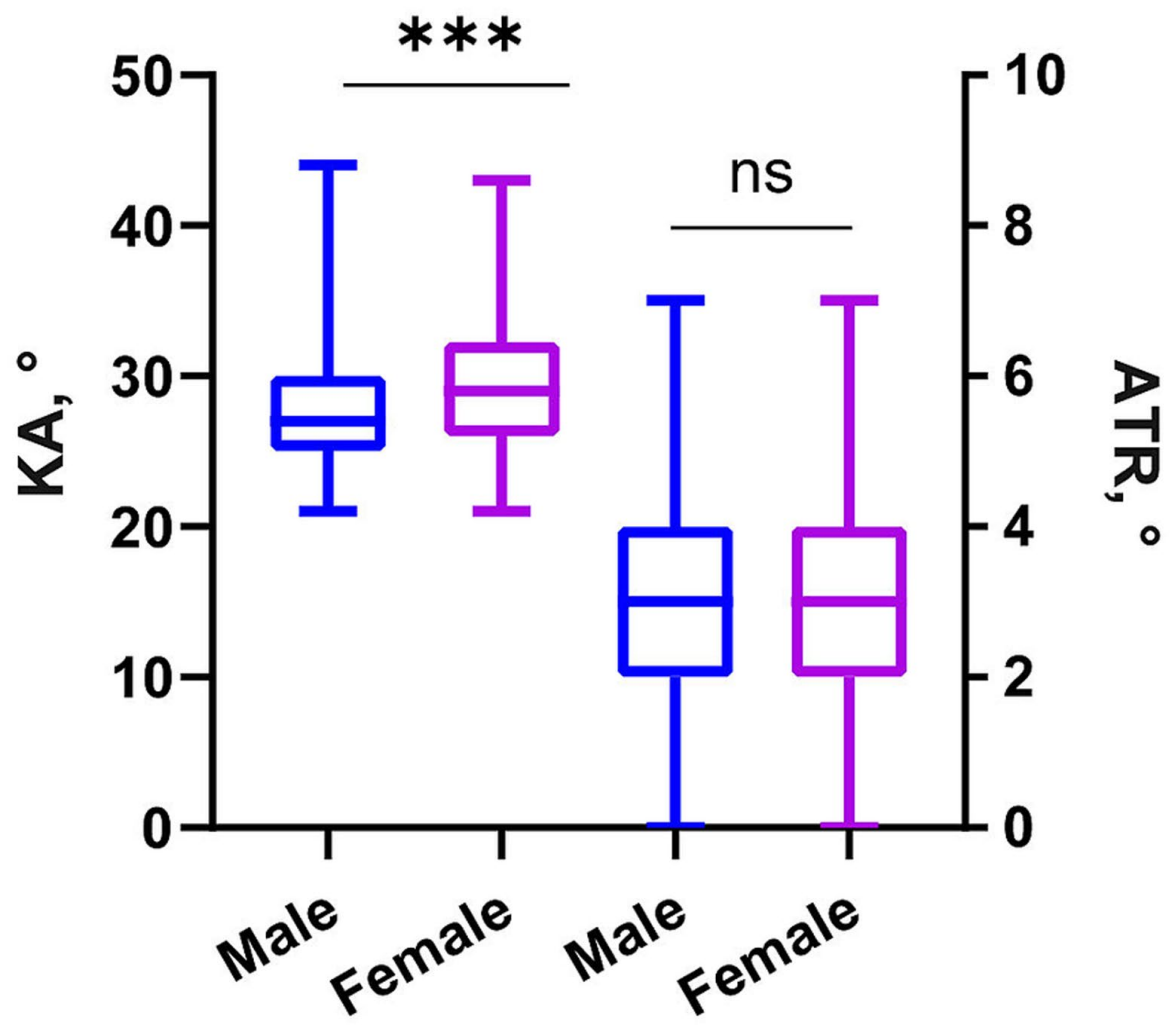
Gender distribution differences in kyphosis angle and ATR. ***p < 0.001, ns, no significance. SE, spherical equivalent. ATR, angle of trunk rotation. KA, kyphosis angle.

In this study, we analyzed the distribution of KA and ATR between male and female participants (Figure 6). Gender differences in spinal parameters were first assessed using the Mann–Whitney U test. A significant difference in KA distribution was observed between males (*n* = 512, median: 27°, IQR: 25°–30°) and females (*n* = 345, median: 29°, IQR: 26°–31°) (*Z* = −4.76, *p* < 0.001), with females showing higher median KA values. No significant gender difference was found for ATR (both medians: 3°, IQR: 2°–4°; *Z* = −0.83, *p* > 0.05).

In the multivariable linear regression model adjusting for age, stereopsis grades, and refractive status, the association between gender and KA was no longer statistically significant (*β* = −0.045, *p* = 0.159), suggesting that the initial gender-related difference in KA may be influenced by other correlated factors (Supplementary Table S2).

## Discussion

In recent years, children’s physical examinations have become increasingly popular in China, aiming to facilitate early detection and intervention of health conditions during children’s growth and development (5). Early detection and intervention are crucial for spinal health and posture. This study explores the relationship between visual function, refractive status, and spinal health in adolescents, focusing on ATR and KA and their associations with visual function and gender.

Stereopsis refers to the ability of the eyes and visual center to perceive depth and distance in three-dimensional space (16, 17). Our results suggest that even minor impairments in stereopsis are associated with altered ATR and spinal health. Specifically, participants with lower stereopsis scores exhibited more pronounced KA, indicating that better visual function correlated with more favorable spinal alignment (18). These findings highlight the importance of maintaining proper visual function to mitigate the progression of spinal deformities.

We also observed a significant correlation between refractive status and spinal abnormalities. Myopic participants showed higher ATR and KA values, supporting previous studies that suggest a relationship between myopia and spinal deformities like scoliosis and kyphosis (19). This underscores the importance of early refractive screenings as part of a comprehensive approach to adolescent health (20). The multivariate regression model adjusting for age, gender confirmed that stereopsis and SE remained independently associated with KA and ATR (Supplementary Tables S1, S2), indicating that the association is not solely driven by confounding factors. The univariable analysis revealed a higher median KA in females compared to males, but this association was not retained in the multivariable model after adjusting for potential confounders such as age, stereopsis, and refractive status. This discrepancy suggests that the observed gender difference in KA may not be a direct, independent effect of gender itself, but rather mediated or masked by other factors. For example, pubertal timing (21–23) (correlated with both gender and spinal development) or gender-specific differences in visual function could contribute to the initial unadjusted association. Thus, while population-level data indicate a gender-related variation in KA, this pattern does not persist as an independent association when accounting for key developmental and visual factors.

This study has several limitations. First, it is a single-center cross-sectional study, and participants were recruited during summer vacation. Many students may leave their local area during this period, potentially reducing the representativeness of the sample. Second, the screening method was not supplemented by radiographic data to confirm spinal abnormalities. And we did not consider lifestyle habits, psychological factors other confounders (24, 25). A potential limitation is the use of habitual refractive correction during stereopsis testing. Previous studies have shown that different refractive correction methods may exert a certain impact on children’s posture and visual function (26, 27).

Also, some scholars have pointed out that there are differences in the innate spinal morphology of children (28). The detailed classification research on spinal morphology is not reflected in this screening-type study. Additionally, the age range of 6–16 years was broad, and further research with refined age stratification would be beneficial. Future research should exclude confounding factors, quantify psychological and lifestyle assessments, define the type of spinal morphology and age range, and track abnormal participants over time to explore the mechanisms between visual and spinal health more thoroughly (25, 29).

## Conclusion

Our cross-sectional study finds that poorer stereopsis, more myopic refractive error, and female sex are associated with modestly higher KA and ATR in adolescents—though the link between female sex and KA may relate to puberty-related changes and needs further confirmation. These observations suggest potential interconnections between visual function, refractive status, and spinal health, implying that including both visual and spinal assessments in routine adolescent screenings could support comprehensive monitoring. The current findings provide preliminary insights, with additional longitudinal studies needed to clarify underlying mechanisms and long-term clinical implications of these associations.

## Data Availability

The original contributions presented in the study are included in the article/Supplementary material, further inquiries can be directed to the corresponding authors.

## Footnotes

1
https://www.gpower.hhu.de/en.html
