# Supplementary material for: The association between spinal health and visual function in a pediatric population: insights from large-scale health examinations
Source: Front Public Health. 2026 Jan 21;13:1702548. doi: 10.3389/fpubh.2025.1702548 (PMC12868225; doi:10.3389/fpubh.2025.1702548)
Supplement: Supplementary file 1 [file Table_1.docx]

Supplementary Table 1. Multivariable Linear Regression Analysis of Factors Associated with KA

| Variable | Unstandardized Coefficient (B) | SE | Standardized Coefficient (β) | t | p-value | VIF |
| --- | --- | --- | --- | --- | --- | --- |
| Model Summary | Adjusted R² = 0.152; F = 25.485; Overall p < 0.001 |  |  |  |  |  |
| (Constant) | 31.152 | 1.110 | - | 28.065 | < 0.001*** | - |
| Visual Function Grade |  |  |  |  |  |  |
| • Grade 0 vs. Grade 2 | -0.959 | 0.097 | -0.323 | -9.897 | < 0.001*** | 1.029 |
| • Grade 1 vs. Grade 2 | -2.062 | 0.414 | -0.173 | -4.980 | < 0.001*** | 1.165 |
| Refractive Status |  |  |  |  |  |  |
| • Right Eye SE (D) | -0.015 | 0.081 | -0.006 | -0.181 | 0.857 | 1.068 |
| Confounders |  |  |  |  |  |  |
| • Sex (Female vs. Male)^1^ | -0.361 | 0.256 | -0.045 | -1.410 | 0.159 | 1.005 |
| • Age (years) | 0.075 | 0.049 | 0.050 | 1.535 | 0.125 | 1.035 |

Notes:

Dependent variable: Kyphosis angle (KA, °).

¹, Sex coded as 1 = Female, 2 = Male.

VIF (Variance Inflation Factor) < 2 indicates no significant multicollinearity.

Significance levels: p < 0.05, p < 0.01, p < 0.001.
