# Supplementary material for: The association between spinal health and visual function in a pediatric population: insights from large-scale health examinations
Source: Front Public Health. 2026 Jan 21;13:1702548. doi: 10.3389/fpubh.2025.1702548 (PMC12868225; doi:10.3389/fpubh.2025.1702548)
Supplement: Supplementary file 2 [file Table_2.docx]

Supplementary Table 2. Multivariable Linear Regression Analysis of Factors Associated with ATR

| Variable | Unstandardized Coefficient (B) | SE | Standardized Coefficient (β) | t | p-value | VIF |
| --- | --- | --- | --- | --- | --- | --- |
| Model Summary | Adjusted R² = 0.191; F = 33.487; Overall p < 0.001 |  |  |  |  |  |
| (Constant) | 3.595 | 0.375 | - | 9.599 | < 0.001*** | - |
| Visual Function Grade |  |  |  |  |  |  |
| • Grade 0 vs. Grade 2 (Reference) | -0.295 | 0.033 | -0.287 | -9.025 | < 0.001*** | 1.029 |
| • Grade 1 vs. Grade 2 (Reference) | -0.059 | 0.140 | -0.014 | -0.422 | 0.673 | 1.166 |
| Right Eye SE (D) | -0.270 | 0.027 | -0.321 | -9.905 | < 0.001*** | 1.068 |
| Confounders |  |  |  |  |  |  |
| Sex (Female vs. Male)^1^ | -0.034 | 0.086 | -0.012 | -0.389 | 0.697 | 1.005 |
| Age (years) | -0.034 | 0.016 | -0.066 | -2.066 | 0.039* | 1.035 |

Notes:

Dependent variable: Trunk Rotation Angle (ATR, °).

¹, Sex coded as 1 = Female, 2 = Male.

VIF (Variance Inflation Factor) < 2 indicates no significant multicollinearity.

Significance levels: p < 0.05, p < 0.01, p < 0.001.
